# *catena*-Poly[[bis­(methanol-κ*O*)bis­(thio­cyanato-κ*N*)cobalt(II)]-μ-1,3-bis­(pyridin-4-yl)propane-κ^2^ *N*,*N*′]

**DOI:** 10.1107/S1600536812000499

**Published:** 2012-01-11

**Authors:** Susanne Wöhlert, Christian Näther

**Affiliations:** a Institut für Anorganische Chemie, Christian-Albrechts-Universität Kiel, Max-Eyth Strasse 2, D-24118 Kiel, Germany

## Abstract

The asymmetric unit of the title compound, [Co(NCS)_2_(C_13_H_14_N_2_)(CH_3_OH)_2_], consists of one cobalt(II) cation located on a center of inversion, one half of a 1,3-bis­(pyridin-4-yl)propane ligand located on a twofold rotation axis, as well as one thio­cyanate anion and one methanol mol­ecule in general positions. The cobalt(II) cation is coordinated by two terminal *N*-bonded thio­cyanate anions and two *N*-bonded 1,3-bis­(pyridin-4-yl)propane ligands, as well as two O atoms of methanol mol­ecules in a slightly distorted octa­hedral coordination mode. Adjacent cations are connected into chains parallel to [10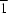] by the bridging 1,3-bis­(pyridin-4-yl)propane ligands. These chains are connected through inter­molecular O—H⋯S hydrogen bonds between the methanol hy­droxy group and the terminal S atom of the thio­cyanate anion.

## Related literature

For related structures, see: Merz *et al.* (2004 ▶). For background literature for this work, see: Boeckmann & Näther (2010 ▶); Wöhlert *et al.* (2011 ▶); Wriedt *et al.* (2009 ▶). For a description of the Cambridge Structural Database, see: Allen (2002 ▶).
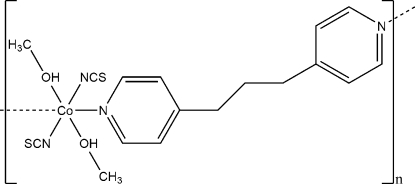


## Experimental

### 

#### Crystal data

[Co(NCS)_2_(C_13_H_14_N_2_)(CH_4_O)_2_]
*M* *_r_* = 437.44
Monoclinic, 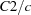

*a* = 20.5440 (12) Å
*b* = 7.5708 (3) Å
*c* = 13.4274 (7) Å
β = 95.176 (5)°
*V* = 2079.91 (18) Å^3^
*Z* = 4
Mo *K*α radiation
μ = 1.04 mm^−1^
*T* = 293 K
0.12 × 0.02 × 0.02 mm

#### Data collection

Stoe IPDS-2 diffractometer
Absorption correction: numerical (*X-SHAPE* and *X-RED32*; Stoe & Cie, 2008 ▶) *T*_min_ = 0.971, *T*_max_ = 0.983
7860 measured reflections
2463 independent reflections
2105 reflections with *I* > 2σ(*I*)
*R*_int_ = 0.021

#### Refinement

*R*[*F* ^2^ > 2σ(*F* ^2^)] = 0.035
*wR*(*F* ^2^) = 0.081
*S* = 1.05
2463 reflections
125 parameters
H atoms treated by a mixture of independent and constrained refinement
Δρ_max_ = 0.25 e Å^−3^
Δρ_min_ = −0.25 e Å^−3^

### 

Data collection: *X-AREA* (Stoe & Cie, 2008 ▶); cell refinement: *X-AREA*; data reduction: *X-AREA*; program(s) used to solve structure: *SHELXS97* (Sheldrick, 2008 ▶); program(s) used to refine structure: *SHELXL97* (Sheldrick, 2008 ▶); molecular graphics: *XP* in *SHELXTL* (Sheldrick, 2008 ▶) and *DIAMOND* (Brandenburg, 2010 ▶); software used to prepare material for publication: XCIF in *SHELXTL*.

## Supplementary Material

Crystal structure: contains datablock(s) I, global. DOI: 10.1107/S1600536812000499/wm2581sup1.cif

Structure factors: contains datablock(s) I. DOI: 10.1107/S1600536812000499/wm2581Isup2.hkl

Additional supplementary materials:  crystallographic information; 3D view; checkCIF report

## Figures and Tables

**Table 1:** Selected bond lengths (Å)

Co1—N1	2.0887 (17)
Co1—O1	2.1372 (15)
Co1—N10	2.1624 (15)

**Table 2:** Hydrogen-bond geometry (Å, °)

*D*—H⋯*A*	*D*—H	H⋯*A*	*D*⋯*A*	*D*—H⋯*A*
O1—H1*O*1⋯S1^i^	0.74 (4)	2.54 (4)	3.2539 (19)	165 (4)

Symmetry code: (i) 
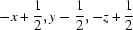
.

**Fig. 1.**
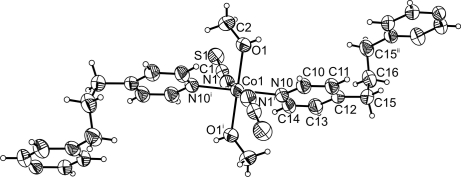
: Crystal structure of the title compound with labelling and displacement ellipsoids drawn at the 50% probability level. [Symmetry codes: i) -x+1/2; -y+1/2; -z+1 and ii) -x; y; -z+1/2.]

**Fig. 2.**
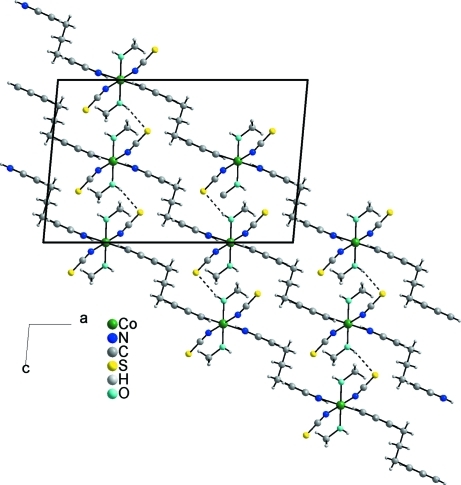
: Crystal structure of the title compound in a view along the b axis. O—H···S hydrogen bonds are depicted with dotted lines.

## supplementary crystallographic information

### Comment

In the last few years we have demonstrated that thermal decomposition reactions are an elegante route for the selective synthesis of new ligand-deficient coordination polymers with cooperative magnetic properties (Boeckmann & Näther, 2010; Wöhlert *et al.*, 2011). In this procedure ligand-rich precursor compounds based on paramagnetic transition metal thiocyanates and neutral monodentate or bidentate *N*-donor ligands are heated, leading to a stepwise loss of the neutral ligands, which yields ligand-deficient coordination compounds (Wriedt *et al.*, 2009). For the preparation of new precursor compounds we have reacted cobalt(II) thiocyanate and 1,3-bis(pyridin-4-yl)-propane in methanol. In this reaction light-green single crystals of the title compound, [Co(NCS)_2_(C_13_H_14_N_2_)(CH_3_OH)_2_], were obtained, which were characterized by single-crystal X-ray diffraction.

In the crystal structure of the title compound the cobalt(II) cations are coordinated by two terminal *N*-bonded thiocyanate anions, two *O*-bonded methanol molecules and two *N*-bonded 1,3-bis(pyridin-4-yl)-propane ligands (Fig. 1). The octahedral coordination sphere of the cobalt(II) cation is slightly distorted with distances in the range of 2.0887 (17) Å to 2.1624 (15) Å. The angles around the cobalt(II) cations range from 88.43 (6) ° to 180 °. The Co(II) cations are bridged by the neutral 1,3-bis(pyridin-4-yl)-propane ligand into chains parallel to [101] (Fig. 2). These chains are further connected through intermolecular O—H···S hydrogen between the methanol molecules and terminal S atoms of the anions (Fig. 2, Table 2). It should be noted that according to a search in the CCDC database (CONQUEST; version 13.2011; Allen, 2002) one structure based on cobalt(II) thiocyanate and 1,3-bis(pyridin-4-yl)-propane has already been reported (Merz *et al.*, 2004). In this structure the cobalt(II) cations are octahedrally coordinated by four 1,3-bis(pyridin-4-yl)-propane ligands and two terminal *N*-bonded thiocyanato anions. The cobalt(II) cations are linked by the 1,3-bis(pyridin-4-yl)-propane ligands into chains oriented along the crystallographic *a*-axis that are further connected by the neutral co-ligands into layers.

### Experimental

Cobalt(II) thiocyanate, 1,3-bis(pyridin-4-yl)-propane and methanol were obtained from Alfa Aesar and were used without further purification. 0.6 mmol (104.4 mg) cobalt(II) thiocyanate, 0.15 mmol (34.4 mg) 1,3-bis(pyridin-4-yl)-propane and 1 mL methanol were reacted in a closed snap-vial without stirring. After the mixture has been standing for several days at room temperature, light-green single crystals suitable for X-ray diffraction were obtained.

### Refinement

The aromatic H atoms were positioned with idealized geometry and were refined isotropically with *U*_eq_(H) = 1.2 *U*_eq_(C) and C—H distances of 0.93 Å using a riding model. The methyl H atoms of the methanol molecule were positioned with idealized geometry and were allowed to rotate but not to tip and were refined isotropically with *U*_eq_(H) = 1.5 *U*_eq_(C) and C—H distances of 0.96 Å using a riding model. The O-H hydrogen atom was located in a difference map and was refined isotropically with varying coordinates.

### Crystal data

**Table:**

[Co(NCS)_2_(C_13_H_14_N_2_)(CH_4_O)_2_]	*F*(000) = 908
*M**_r_* = 437.44	*D*_x_ = 1.397 Mg m^−^^3^
Monoclinic, *C*2/*c*	Mo *K*α radiation, λ = 0.71073 Å
Hall symbol: -C 2yc	Cell parameters from 7860 reflections
*a* = 20.5440 (12) Å	θ = 2.9–28.0°
*b* = 7.5708 (3) Å	µ = 1.04 mm^−^^1^
*c* = 13.4274 (7) Å	*T* = 293 K
β = 95.176 (5)°	Block, light-green
*V* = 2079.91 (18) Å^3^	0.12 × 0.02 × 0.02 mm
*Z* = 4	

### Data collection

**Table:**

Stoe IPDS-2 diffractometer	2463 independent reflections
Radiation source: fine-focus sealed tube	2105 reflections with *I* > 2σ(*I*)
graphite	*R*_int_ = 0.021
ω scans	θ_max_ = 28.0°, θ_min_ = 2.9°
Absorption correction: numerical (*X-SHAPE* and *X-RED32*; Stoe & Cie, 2008)	*h* = −26→26
*T*_min_ = 0.971, *T*_max_ = 0.983	*k* = −9→9
7860 measured reflections	*l* = −17→17

### Refinement

**Table:**

Refinement on *F*^2^	Primary atom site location: structure-invariant direct methods
Least-squares matrix: full	Secondary atom site location: difference Fourier map
*R*[*F*^2^ > 2σ(*F*^2^)] = 0.035	Hydrogen site location: inferred from neighbouring sites
*wR*(*F*^2^) = 0.081	H atoms treated by a mixture of independent and constrained refinement
*S* = 1.05	*w* = 1/[σ^2^(*F*_o_^2^) + (0.0342*P*)^2^ + 1.3966*P*] where *P* = (*F*_o_^2^ + 2*F*_c_^2^)/3
2463 reflections	(Δ/σ)_max_ < 0.001
125 parameters	Δρ_max_ = 0.25 e Å^−^^3^
0 restraints	Δρ_min_ = −0.25 e Å^−^^3^

### Special details

**Table:**

Geometry. All esds (except the esd in the dihedral angle between two l.s. planes) are estimated using the full covariance matrix. The cell esds are taken into account individually in the estimation of esds in distances, angles and torsion angles; correlations between esds in cell parameters are only used when they are defined by crystal symmetry. An approximate (isotropic) treatment of cell esds is used for estimating esds involving l.s. planes.
Refinement. Refinement of F^2^ against ALL reflections. The weighted R-factor wR and goodness of fit S are based on F^2^, conventional R-factors R are based on F, with F set to zero for negative F^2^. The threshold expression of F^2^ > 2sigma(F^2^) is used only for calculating R-factors(gt) etc. and is not relevant to the choice of reflections for refinement. R-factors based on F^2^ are statistically about twice as large as those based on F, and R- factors based on ALL data will be even larger.

### Fractional atomic coordinates and isotropic or equivalent isotropic displacement parameters (Å^2^)

**Table:**

	*x*	*y*	*z*	*U*_iso_*/*U*_eq_	Occ. (<1)
Co1	0.2500	0.2500	0.5000	0.04506 (12)	
N1	0.30569 (8)	0.4522 (2)	0.44450 (13)	0.0576 (4)	
C1	0.33565 (9)	0.5317 (3)	0.39083 (14)	0.0489 (4)	
S1	0.37746 (3)	0.64359 (9)	0.31502 (4)	0.06640 (17)	
N10	0.16009 (7)	0.3899 (2)	0.45687 (11)	0.0487 (4)	
C10	0.10533 (9)	0.3059 (3)	0.42263 (15)	0.0539 (4)	
H10	0.1051	0.1831	0.4236	0.065*	
C11	0.04904 (9)	0.3927 (3)	0.38589 (15)	0.0574 (5)	
H11	0.0123	0.3283	0.3625	0.069*	
C12	0.04733 (9)	0.5744 (3)	0.38384 (14)	0.0525 (5)	
C13	0.10384 (10)	0.6616 (3)	0.42123 (17)	0.0607 (5)	
H13	0.1050	0.7844	0.4224	0.073*	
C14	0.15805 (10)	0.5664 (3)	0.45640 (16)	0.0568 (5)	
H14	0.1952	0.6279	0.4812	0.068*	
C15	−0.01223 (10)	0.6752 (4)	0.34160 (16)	0.0639 (6)	
H15A	−0.0263	0.7532	0.3928	0.077*	
H15B	−0.0474	0.5923	0.3238	0.077*	
C16	0.0000	0.7841 (4)	0.2500	0.0652 (8)	
H16A	0.0375	0.8597	0.2666	0.078*	0.50
H16B	−0.0375	0.8597	0.2334	0.078*	0.50
O1	0.24410 (8)	0.1222 (3)	0.35756 (11)	0.0623 (4)	
H1O1	0.2117 (13)	0.118 (4)	0.328 (2)	0.076 (9)*	
C2	0.29545 (11)	0.1156 (4)	0.29272 (18)	0.0723 (7)	
H2A	0.2931	0.2178	0.2502	0.108*	
H2B	0.2909	0.0109	0.2524	0.108*	
H2C	0.3369	0.1138	0.3319	0.108*	

### Atomic displacement parameters (Å^2^)

**Table:**

	*U*^11^	*U*^22^	*U*^33^	*U*^12^	*U*^13^	*U*^23^
Co1	0.03980 (18)	0.0573 (2)	0.03795 (17)	−0.00119 (15)	0.00289 (12)	0.00289 (15)
N1	0.0515 (9)	0.0658 (11)	0.0553 (9)	−0.0043 (8)	0.0043 (7)	0.0111 (8)
C1	0.0442 (9)	0.0541 (10)	0.0472 (9)	0.0013 (8)	−0.0023 (7)	0.0005 (8)
S1	0.0598 (3)	0.0797 (4)	0.0602 (3)	−0.0151 (3)	0.0084 (2)	0.0112 (3)
N10	0.0408 (7)	0.0627 (10)	0.0423 (8)	0.0013 (7)	0.0013 (6)	0.0001 (7)
C10	0.0463 (10)	0.0645 (12)	0.0506 (10)	−0.0046 (8)	0.0018 (8)	0.0022 (9)
C11	0.0423 (9)	0.0770 (14)	0.0522 (10)	−0.0071 (9)	0.0002 (8)	0.0007 (10)
C12	0.0426 (9)	0.0757 (13)	0.0391 (9)	0.0062 (9)	0.0037 (7)	−0.0040 (9)
C13	0.0564 (11)	0.0602 (13)	0.0633 (12)	0.0075 (10)	−0.0061 (9)	−0.0088 (10)
C14	0.0473 (10)	0.0624 (12)	0.0585 (11)	−0.0006 (9)	−0.0072 (8)	−0.0075 (10)
C15	0.0471 (10)	0.0889 (16)	0.0552 (11)	0.0141 (10)	0.0010 (9)	−0.0059 (11)
C16	0.0563 (16)	0.066 (2)	0.0705 (19)	0.000	−0.0128 (14)	0.000
O1	0.0474 (8)	0.0938 (12)	0.0454 (7)	0.0011 (8)	0.0029 (6)	−0.0106 (8)
C2	0.0583 (12)	0.1018 (19)	0.0582 (12)	0.0048 (12)	0.0135 (10)	−0.0160 (13)

### Geometric parameters (Å, °)

**Table:**

Co1—N1	2.0887 (17)	C12—C15	1.509 (3)
Co1—N1^i^	2.0887 (17)	C13—C14	1.374 (3)
Co1—O1	2.1372 (15)	C13—H13	0.9300
Co1—O1^i^	2.1372 (15)	C14—H14	0.9300
Co1—N10	2.1624 (15)	C15—C16	1.520 (3)
Co1—N10^i^	2.1624 (15)	C15—H15A	0.9700
N1—C1	1.158 (2)	C15—H15B	0.9700
C1—S1	1.628 (2)	C16—C15^ii^	1.520 (3)
N10—C14	1.337 (3)	C16—H16A	0.9700
N10—C10	1.337 (2)	C16—H16B	0.9700
C10—C11	1.382 (3)	O1—C2	1.428 (2)
C10—H10	0.9300	O1—H1O1	0.74 (3)
C11—C12	1.376 (3)	C2—H2A	0.9600
C11—H11	0.9300	C2—H2B	0.9600
C12—C13	1.390 (3)	C2—H2C	0.9600
			
N1—Co1—N1^i^	180.00 (10)	C13—C12—C15	121.2 (2)
N1—Co1—O1	90.07 (7)	C14—C13—C12	120.0 (2)
N1^i^—Co1—O1	89.93 (7)	C14—C13—H13	120.0
N1—Co1—O1^i^	89.93 (7)	C12—C13—H13	120.0
N1^i^—Co1—O1^i^	90.07 (7)	N10—C14—C13	123.36 (19)
O1—Co1—O1^i^	180.0	N10—C14—H14	118.3
N1—Co1—N10	91.57 (6)	C13—C14—H14	118.3
N1^i^—Co1—N10	88.43 (6)	C12—C15—C16	113.01 (16)
O1—Co1—N10	90.23 (6)	C12—C15—H15A	109.0
O1^i^—Co1—N10	89.77 (6)	C16—C15—H15A	109.0
N1—Co1—N10^i^	88.43 (6)	C12—C15—H15B	109.0
N1^i^—Co1—N10^i^	91.57 (6)	C16—C15—H15B	109.0
O1—Co1—N10^i^	89.77 (6)	H15A—C15—H15B	107.8
O1^i^—Co1—N10^i^	90.23 (6)	C15—C16—C15^ii^	114.3 (3)
N10—Co1—N10^i^	180.00 (8)	C15—C16—H16A	108.7
C1—N1—Co1	160.42 (17)	C15^ii^—C16—H16A	108.7
N1—C1—S1	179.7 (2)	C15—C16—H16B	108.7
C14—N10—C10	116.65 (17)	C15^ii^—C16—H16B	108.7
C14—N10—Co1	121.10 (13)	H16A—C16—H16B	107.6
C10—N10—Co1	122.08 (14)	C2—O1—Co1	125.17 (14)
N10—C10—C11	123.2 (2)	C2—O1—H1O1	110 (2)
N10—C10—H10	118.4	Co1—O1—H1O1	118 (2)
C11—C10—H10	118.4	O1—C2—H2A	109.5
C12—C11—C10	120.10 (19)	O1—C2—H2B	109.5
C12—C11—H11	120.0	H2A—C2—H2B	109.5
C10—C11—H11	120.0	O1—C2—H2C	109.5
C11—C12—C13	116.67 (18)	H2A—C2—H2C	109.5
C11—C12—C15	122.1 (2)	H2B—C2—H2C	109.5

Symmetry codes: (i) −*x*+1/2, −*y*+1/2, −*z*+1; (ii) −*x*, *y*, −*z*+1/2.

### Hydrogen-bond geometry (Å, °)

**Table:**

*D*—H···*A*	*D*—H	H···*A*	*D*···*A*	*D*—H···*A*
O1—H1O1···S1^iii^	0.74 (4)	2.54 (4)	3.2539 (19)	165 (4)

Symmetry codes: (iii) −*x*+1/2, *y*−1/2, −*z*+1/2.
